# Increased predominance of the matured ventricular subtype in embryonic stem cell-derived cardiomyocytes in vivo

**DOI:** 10.1038/s41598-020-68373-9

**Published:** 2020-07-17

**Authors:** Hajime Ichimura, Shin Kadota, Toshihide Kashihara, Mitsuhiko Yamada, Kuniaki Ito, Hideki Kobayashi, Yuki Tanaka, Naoko Shiba, Shinichiro Chuma, Shugo Tohyama, Tatsuichiro Seto, Kenji Okada, Koichiro Kuwahara, Yuji Shiba

**Affiliations:** 1 Department of Regenerative Science and Medicine, Shinshu University School of Medicine, Matsumoto, Japan; 2 Institute for Biomedical Sciences, Shinshu University, Matsumoto, Japan; 3 Division of Cardiovascular Surgery, Department of Surgery, Shinshu University School of Medicine, Matsumoto, Japan; 4 Department of Cell Biology and Molecular Medicine, Cardiovascular Research Institute, Rutgers New Jersey Medical School, Newark, USA; 5 Department of Molecular Pharmacology, Shinshu University School of Medicine, Matsumoto, Japan; 6 Department of Cardiovascular Medicine, Shinshu University School of Medicine, Matsumoto, Japan; 7 Department of Pediatrics, Shinshu University School of Medicine, Matsumoto, Japan; 8 Department of Development and Differentiation, Institute for Frontier Life and Medical Sciences, Kyoto University, Kyoto, Japan; 9 Department of Cardiology, Keio University School of Medicine, Tokyo, Japan; 10 Department of Surgery, Division of Cardiovascular Surgery, Kobe University Graduate School of Medicine, Kobe, Japan

**Keywords:** Embryonic stem cells, Regeneration

## Abstract

Accumulating evidence suggests that human pluripotent stem cell-derived cardiomyocytes can affect “heart regeneration”, replacing injured cardiac scar tissue with concomitant electrical integration. However, electrically coupled graft cardiomyocytes were found to innately induce transient post-transplant ventricular tachycardia in recent large animal model transplantation studies. We hypothesised that these phenomena were derived from alterations in the grafted cardiomyocyte characteristics. In vitro experiments showed that human embryonic stem cell-derived cardiomyocytes (hESC-CMs) contain nodal-like cardiomyocytes that spontaneously contract faster than working-type cardiomyocytes. When transplanted into athymic rat hearts, proliferative capacity was lower for nodal-like than working-type cardiomyocytes with grafted cardiomyocytes eventually comprising only relatively matured ventricular cardiomyocytes. RNA-sequencing of engrafted hESC-CMs confirmed the increased expression of matured ventricular cardiomyocyte-related genes, and simultaneous decreased expression of nodal cardiomyocyte-related genes. Temporal engraftment of electrical excitable nodal-like cardiomyocytes may thus explain the transient incidence of post-transplant ventricular tachycardia, although further large animal model studies will be required to control post-transplant arrhythmia.

## Introduction

Pluripotent stem cells are attractive cell sources for regenerative medicine to treat refractory diseases including heart failure. As adult cardiomyocytes have extremely limited capacity to proliferate^1^, necrotic cardiomyocytes resulting from cardiac injury, such as myocardial infarction, will no longer spontaneously regenerate and are replaced with non-contractile scar tissue, eventually leading to heart failure. To regenerate the heart, transplantation studies of human pluripotent stem cell-derived cardiomyocytes (hPSC-CMs) were performed initially in small animal models, in which human embryonic stem cell-derived cardiomyocytes (hESC-CMs) engrafted and survived in the injured heart^2^, restored contractile function^3,4^, and electrically integrated with host cardiomyocytes^5,6^. In these and other small animal studies^7^, ventricular arrhythmia caused by the transplantation of hPSC-CMs was not detected, likely owing to the much faster heart rate of the host species than that of graft cardiomyocytes.

Successful engraftment of electrical integrated hESC-CM grafts in a non-human primate model of myocardial infarction was further reported by Chong et al.^8^. They observed transient ventricular tachycardia (VT) and accelerated idioventricular rhythm in recipients of hESC-CMs. Subsequently, our group generated induced pluripotent stem cell (iPSC)-CMs of the cynomolgus monkey and transplanted these into a monkey model of myocardial infarction in an allogeneic manner^9^. We observed partial remuscularisation of scar tissue at 12 weeks post transplantation in all five recipients along with partial restoration of cardiac contractile function. However, the incidence of VT (both sustained and non-sustained) was significantly increased in the recipients of iPSC-CMs compared to those receiving vehicle. The post-transplant sustained VT was observed in all five recipients of iPSC-CMs at 2 weeks post transplantation; however, the incidence and duration of VT decreased over time and no sustained VT was observed at 12 weeks post transplantation^9^.

In a follow-up to the Chong et al.^8^ study, Liu et al.^10^ and, independently, Romagnuolo et al.^11^ presented mechanistic insights regarding the observed post-transplant arrhythmia. Specifically, they observed transient post-transplant VT in a simian or porcine transplantation model, respectively, and concluded that focal electrical activity, such as evinced in the automaticity of grafted cardiomyocytes, rather than reentrant mechanism, constituted the mechanism underlying the post-transplant VT as determined by electro-anatomical mapping studies^10,11^. Nevertheless, the factors regulating the transient appearance of post-transplant VT remain unresolved. To date, all reported post-transplant VT has been observed only within a few weeks following cell transplantation^8–11^. Given that the mechanism of post-transplant VT is likely focal graft activity, we hypothesised that the characteristics of the grafted cardiomyocytes might be altered over time. To address this issue, in the present study we transplanted hESC-CMs into the athymic rat heart and compared the characteristics of grafted cardiomyocytes at 2, 4, and 12 weeks post transplantation via histological and bioinformatics analyses.

## Results

### hESC-CMs consist of nodal-like and working-type cardiomyocytes

**Figure 1:**
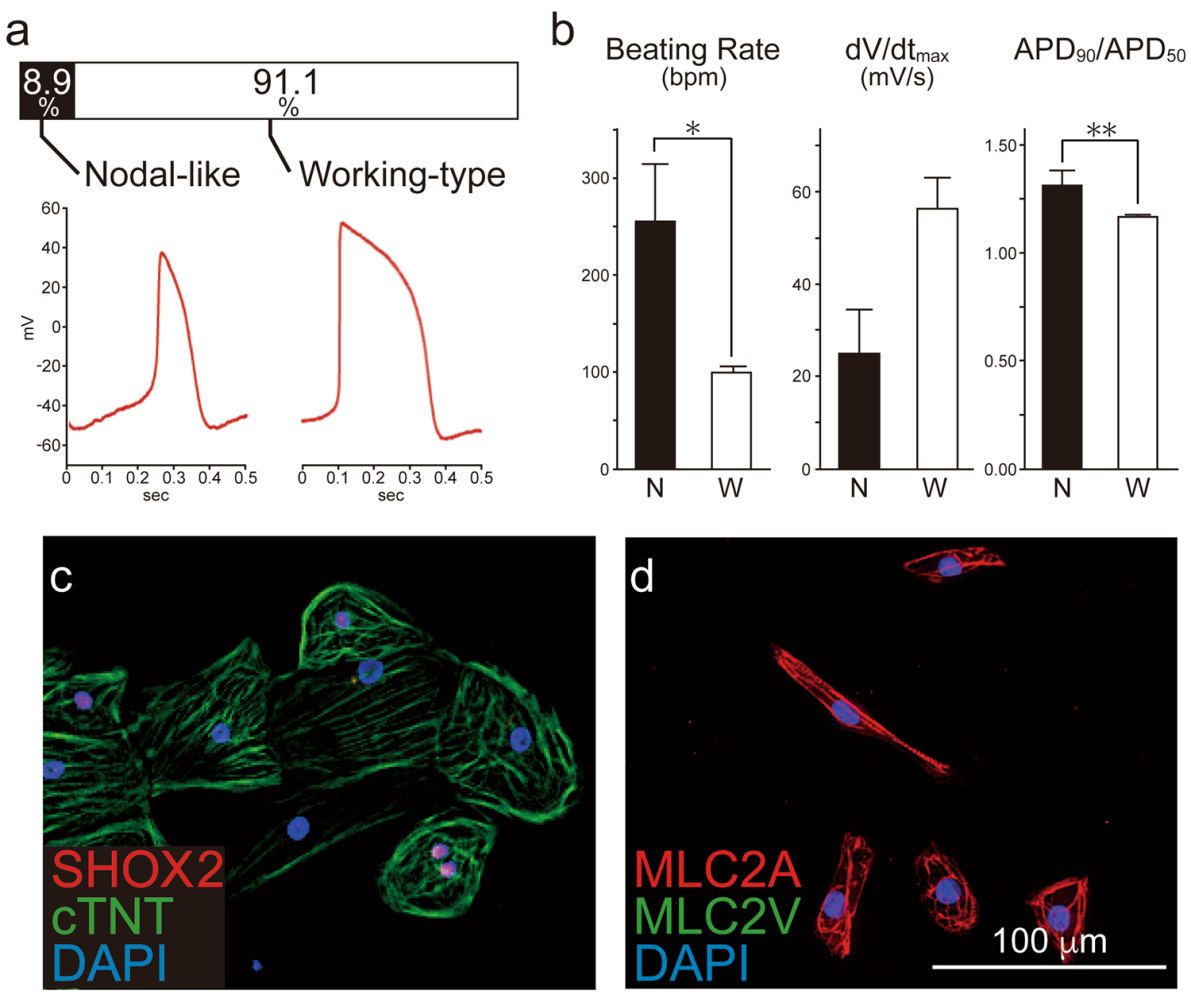
Subtypes of human ES cell-derived cardiomyocytes (hESC-CMs). Undifferentiated human ES cells were transdifferentiated into cardiomyocytes. (**a**) On day 20 after differentiation, hESC-CMs (n = 45) were patch-clamped to record action potential (AP) pattern. Two distinct AP patterns were observed in hESC-CMs; nodal-like and working-type cardiomyocytes. (**b**) Electrophysiological properties of nodal-like (N) and working-type cardiomyocytes (W). Data are mean ± SEM. *P < 0.0001 by Student’s *t* test, **P = 0.0002 by Student’s *t* test. (**c**,**d**) Expression of the nodal marker SHOX2 and cardiac marker cardiac troponin T (cTNT); MLC2A and MLC2V in hESC-CMs on day 20. See also SI Figures S1 and S2.

hESC-CMs were generated using a previously reported direct differentiation protocol^12^, harvested on day 20 after activation, and cryopreserved for both in vitro and in vivo experiments (SI Fig. S1 online). The cardiac purity was 98.7% as determined by flow cytometry with staining against cardiac troponin T (cTNT) (SI Fig. S2 online). Cardiomyocytes were thawed and used as required from the same batch of hESC-CMs in all experiments in this study. We first characterised the spontaneous action potential (AP) patterns of in vitro hESC-CMs. We patch-clamped isolated hESC-CMs and observed largely two distinct AP configurations: 8.9% nodal-like and 91.1% working-type cardiomyocytes (Fig. 1a). The nodal-like cardiomyocytes exhibited significantly faster spontaneous contraction rate, slower AP upstroke, and faster repolarisation than working-type cardiomyocytes (Fig. 1b). Immunocytochemical analysis revealed that 12.5% of cardiomyocytes were positive for the nodal marker SHOX2 and 87.5% were negative (Fig. 1c). The majority of hESC-CMs did not express MLC2V but rather were positive for MLC2A (Fig. 1d), indicating cardiomyocyte immaturity.

### Grafted hESC-CMs grow and become mature over time

**Figure 2:**
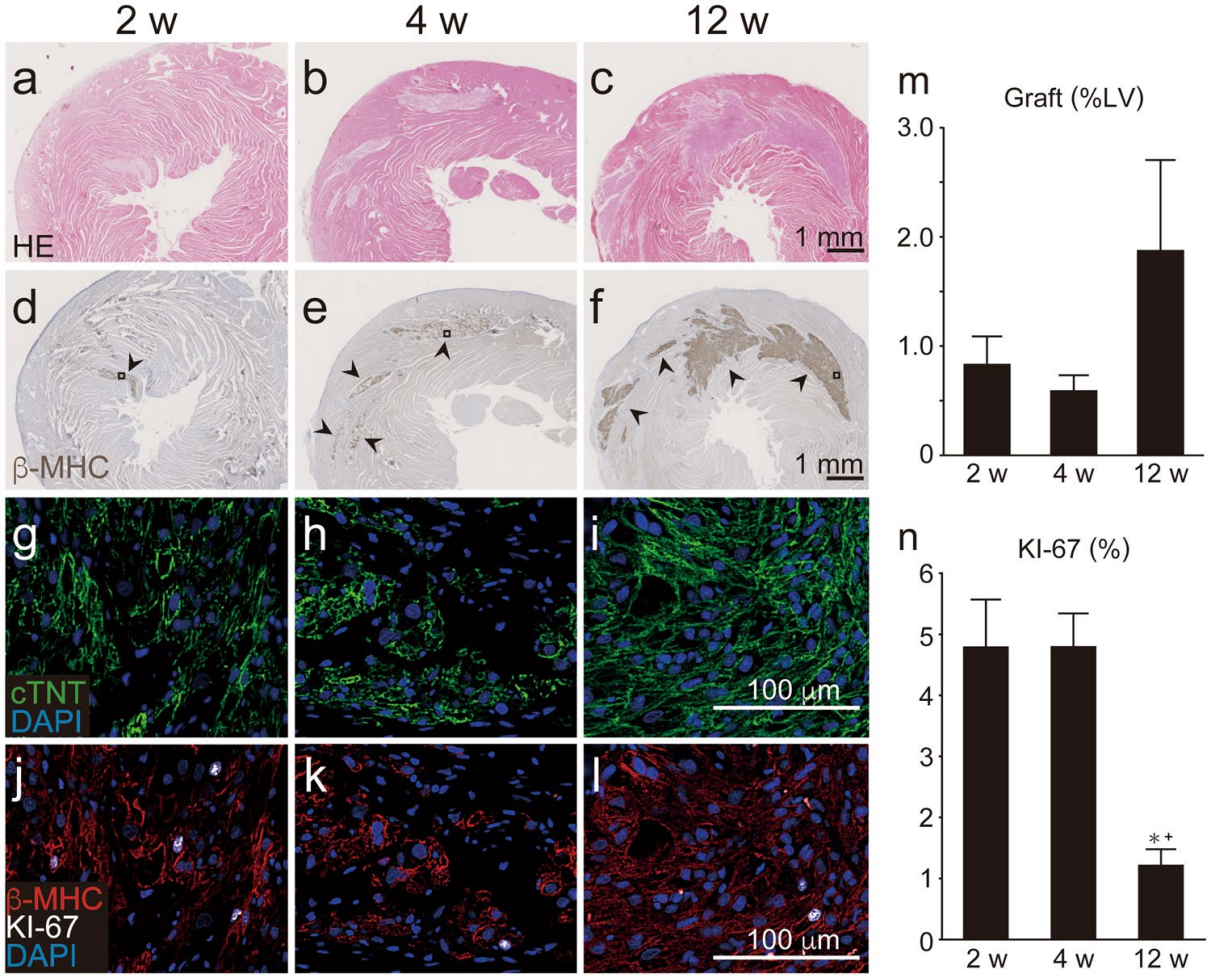
Engraftment of human ES cell-derived cardiomyocytes (hESC-CMs) in athymic rat hearts. hESC-CMs were injected directly into the athymic rat heart and histological analysis was performed at 2, 4, or 12 weeks after transplantation. (**a**–**c**) Hematoxylin–eosin (H&E) staining of grafted hESC-CMs in the host hearts. (**d**–**f**) Human grafted cardiomyocytes identified by the cardiac marker, β-myosin heavy chain (β-MHC, arrowheads). Note that β-MHC was exclusively expressed in grafted human cardiomyocytes but not in host rat cardiomyocytes. Black squares indicate the area where pictures below (**g**–**l**) were taken from. (**g**–**l**) Quadruple staining against cTNT (green), β-MHC (red), KI-67 (white), and DAPI (blue) in the graft area. Grafted cardiomyocytes at 12 weeks post transplantation (**i**,**l**) showed a mature appearance characterised by formation of an aligned sarcomere structure and serried deposition of cardiomyocytes, as well as rare expression of the proliferative marker KI-67. (**m**) Percentage of grafted area divided by total left-ventricular area (n = 5 per group). *LV* left ventricle. (**n**) Percentage of KI-67 positive graft cells at 2, 4, and 12 weeks post transplantation. *P = 0.0019 vs. 2 weeks, + P = 0.0019 vs. 4 weeks by ANOVA with Tukey’s post hoc test. See also SI Figure S3.

To evaluate the in vivo chronological characteristics of hESC-CMs, we transplanted hESC-CMs into the athymic rat heart and harvested the hearts at 2 (2 weeks; n = 5), 4 (4 weeks; n = 5), or 12 (12 weeks; n = 5) weeks post transplantation. These endpoints were designed based on our previous transplantation study in which post-transplant arrhythmia was frequently observed between 2 and 4 weeks whereas no sustained VT was detected at 12 weeks post transplantation^9^. All of the recipients sacrificed at 2, 4, and 12 weeks showed surviving grafts without apparent infiltration of inflammatory cells (Fig. 2a–c). Graft tissue exclusively consisted of cardiomyocytes as determined by the cardiac specific markers β-myosin heavy chain (β-MHC, Fig. 2d–f) and cTNT (Fig. 2g–i). Grafted cardiomyocytes at 12 weeks post transplantation often showed a clear sarcomere and were arranged in a more serried manner and aligned (Fig. 2g–i). Moreover, co-staining against β-MHC, and the proliferation marker KI-67, demonstrated that graft cardiomyocytes retained substantial proliferative capacity up to 4 weeks following transplantation, however, the proliferative capacity was significantly decreased by 12 weeks (Fig. 2j–l,n). * The fraction of MLC2A-positive cardiomyocytes, which reflects either atrial, nodal, or immature ventricular cells, was significantly decreased at 12 weeks post transplantation compared to that at 2 or 4 weeks post transplantation. In contrast, the fraction of MLC2V-positive mature ventricular cells was significantly increased at 12 weeks post transplantation (SI Fig. S3i–l online). The recipients sacrificed at 12 weeks post transplantation tended to show a larger graft area, although this difference did not reach statistical significance (Fig. 2m). Taken together, the results of histological analyses indicated that the grafted cardiomyocytes grew and became mature in vivo, consistent with previous reports^8,13,14^.

### Transient engraftment of nodal-like cardiomyocytes in vivo

**Figure 3:**
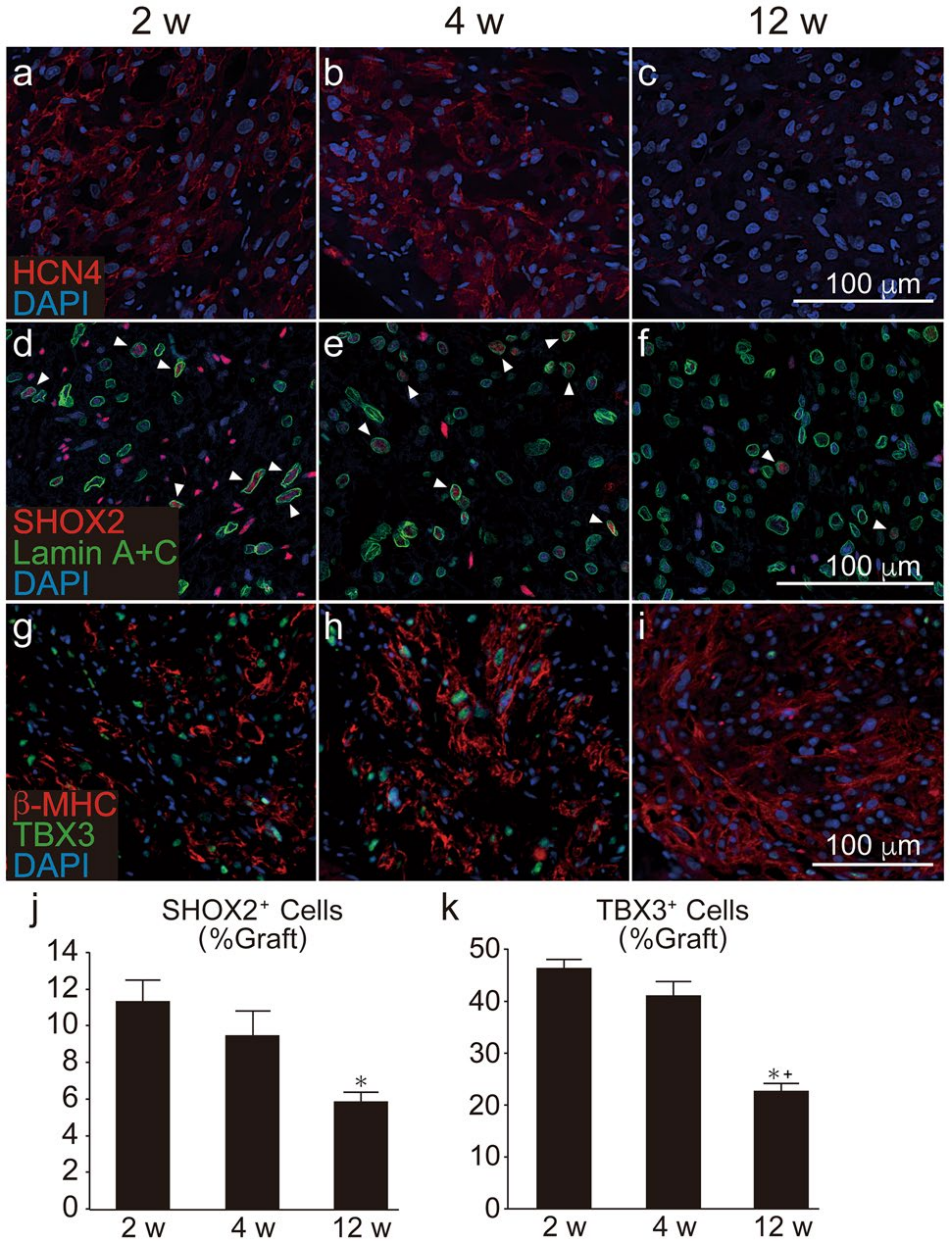
Chronological expression of nodal markers in grafted human ES cell-derived cardiomyocytes (hESC-CMs) in intact hearts. (**a**–**c**) HCN4 (red) staining in the graft area. (**d**–**f**) SHOX2 (red) and human specific Lamin A+C (green) staining. Note that SHOX2 was expressed not only in graft cells but also in host cells, such as fibroblasts. As such, we designated SHOX2^+^/Lamin A+C^+^ cells (arrow heads) as graft nodal cells. (**g**–**i**) Staining against β-MHC (red) and TBX3 (green). (**j**) Quantitative analysis of the number of SHOX2^+^/Lamin A+C^+^ cells divided by the number of total Lamin A+C^+^ graft cells (n = 5 per group). Data are mean ± SEM. *P = 0.0135 vs. 2 weeks by ANOVA with Tukey’s post hoc test. (**k**) Quantitative analysis of the number of TBX3^+^ cells divided by that of β-MHC^+^ graft cardiomyocytes (n = 5 per group). Data are mean ± SEM. *P < 0.0001 vs. 2 weeks, + P = 0.0002 vs. 4 weeks by ANOVA with Tukey’s post hoc test.

We next traced the nodal-like cardiomyocytes in grafted tissue by histology. As no perfectly specific antigen for nodal cardiomyocytes has yet been identified to our knowledge, we used three antibodies against HCN4, SHOX2, and TBX3 to trace nodal-like grafted cardiomyocytes. The expression of the pacemaker channel HCN4 in the graft was substantially decreased at 12 weeks post transplantation (Fig. 3a–c). Similarly, the fractions of cells expressing SHOX2 and TBX3 were significantly decreased at 12 weeks post transplantation (Fig. 3d–k).

**Figure 4:**
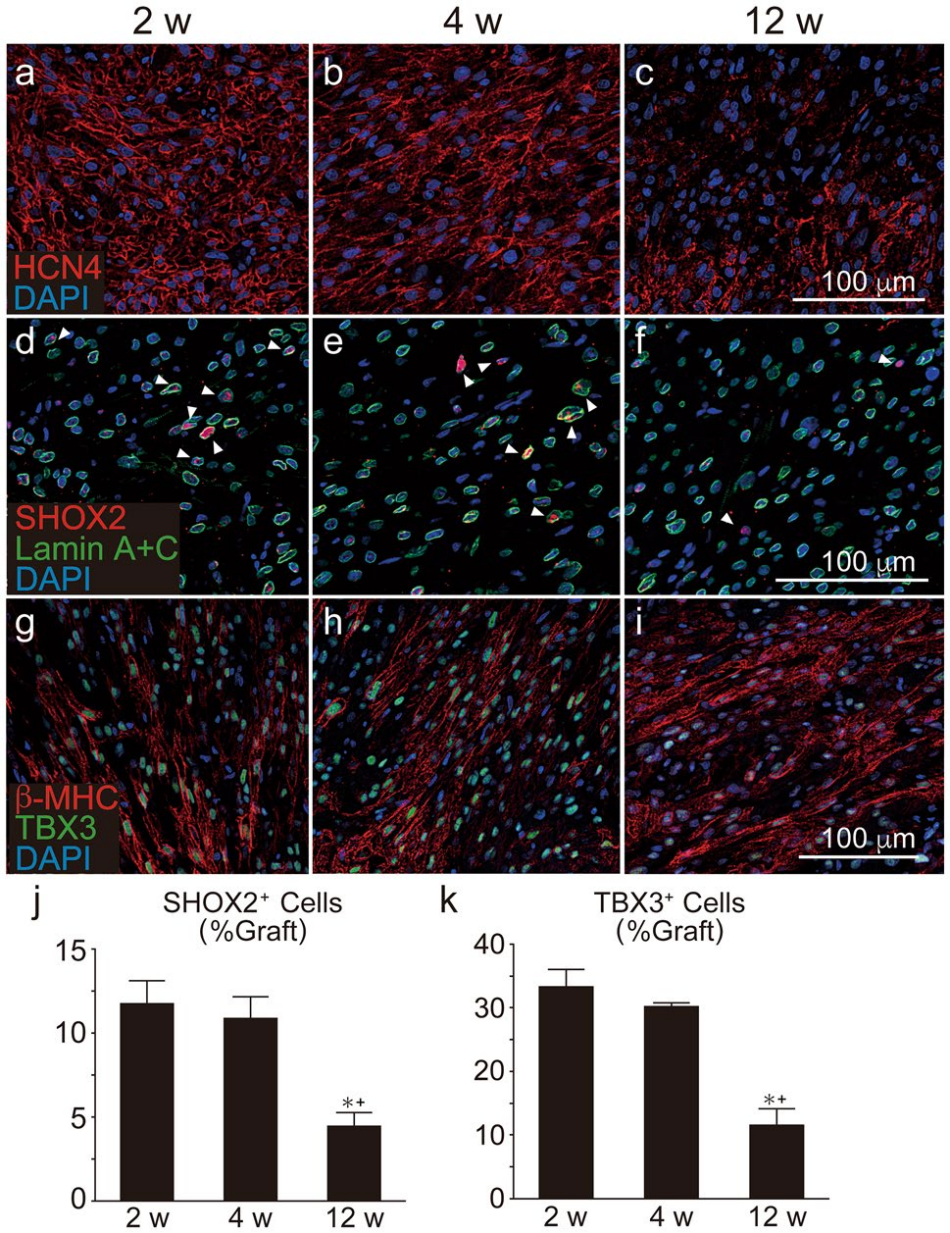
Chronological expression of nodal markers in grafted human ES cell-derived cardiomyocytes (hESC-CMs) in injured hearts. hESC-CMs were transplanted on week after induction of myocardial infarction. Histological analysis was performed at the same time-points. (**a**–**c**) HCN4 (red) staining in the graft area. (**d**–**f**) SHOX2 (red) and human specific Lamin A+C (green) staining. SHOX2^+^/Lamin A+C^+^ cells (arrow heads) were designated as graft nodal cells. (**g**–**i**) Staining against β-MHC (red) and TBX3 (green). (**j**) Quantitative analysis of the number of SHOX2^+^/Lamin A+C^+^ cells divided by the number of total Lamin A+C^+^ graft cells (n = 5 per group). Data represent mean ± SEM. *P = 0.0036 vs. 2 weeks, + P = 0.0116 vs. 4 weeks by ANOVA with Tukey’s post hoc test. (**k**) Quantitative analysis of the number of TBX3 + cells divided by that of β-MHC^+^ graft cardiomyocytes (n = 5 per group). Data represent mean ± SEM. *P = 0.0003 vs. 2 weeks, + P = 0.0015 vs. 4  weeks by ANOVA with Tukey’s post hoc test.

To observe the characteristics of grafted hESC-CMs in injured heart tissue, we created a myocardial infarction model and transplanted hESC-CMs one week after induction of myocardial infarction. All recipients had surviving grafted cardiomyocytes around the injured area (SI Fig. S4 online). As shown in SI Fig. S5 online, the recipients of hESC-CMs tended to exhibit better contractile function by echocardiography compared to the vehicle control, however, the difference did not reach statistical significance. Likewise, in the intact hearts, the expressions of nodal markers HCN4, SHOX2, and TBX3 in the grafted hESC-CMs decreased over time in injured hearts (Fig. 4a–k). These data strongly suggested that transplanted nodal-like cardiomyocytes survived for 4 weeks after transplantation but decreased over the long-term.

### Chronological alteration in hESC-CM characteristics in vitro

**Figure 5:**
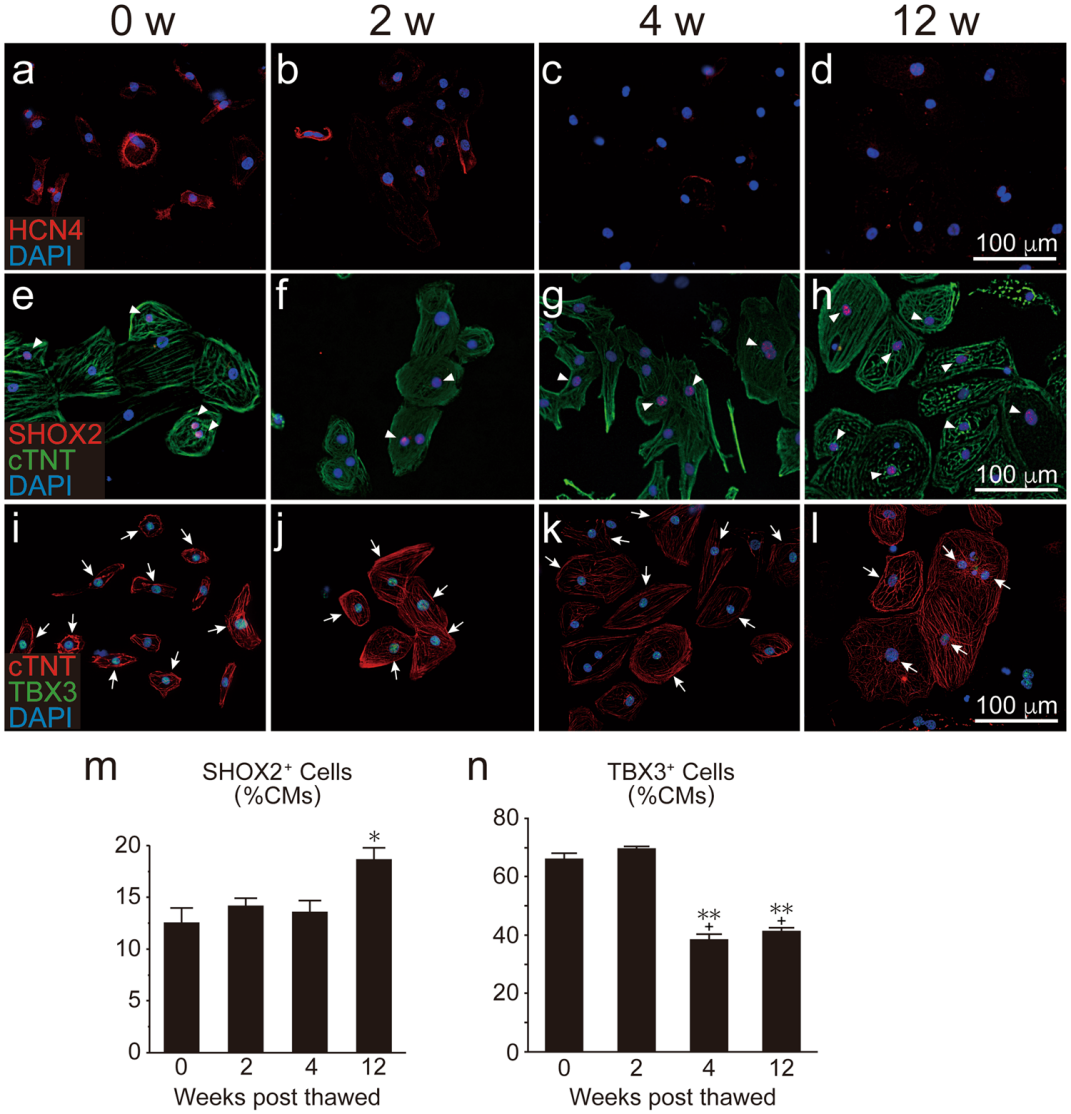
Chronological expression of nodal markers in human ES cell-derived cardiomyocytes (hESC-CMs) on two-dimensional culture. Expression of nodal markers in hESC-CMs. Note that time scales in weeks correspond to those of the in vivo study depicted in Fig. 3. (**a**–**d**) HCN4 (red). (**e–h**) SHOX2 (red, arrowheads) and cTNT (green). (**i**–**l**) cTNT (red) and TBX3 (green, arrows). (**m**,**n**) Fractions of SHOX2^+^ and TBX3^+^ cardiomyocytes, respectively (n = 3 per group). Data are mean ± SEM. *P = 0.026 vs. 0 week by ANOVA with Tukey’s post hoc test in (**m**). **P < 0.0001 vs. 0 week, + P < 0.0001 vs. 2 weeks by ANOVA with Tukey’s post hoc test in (**n**).

**Figure 6:**
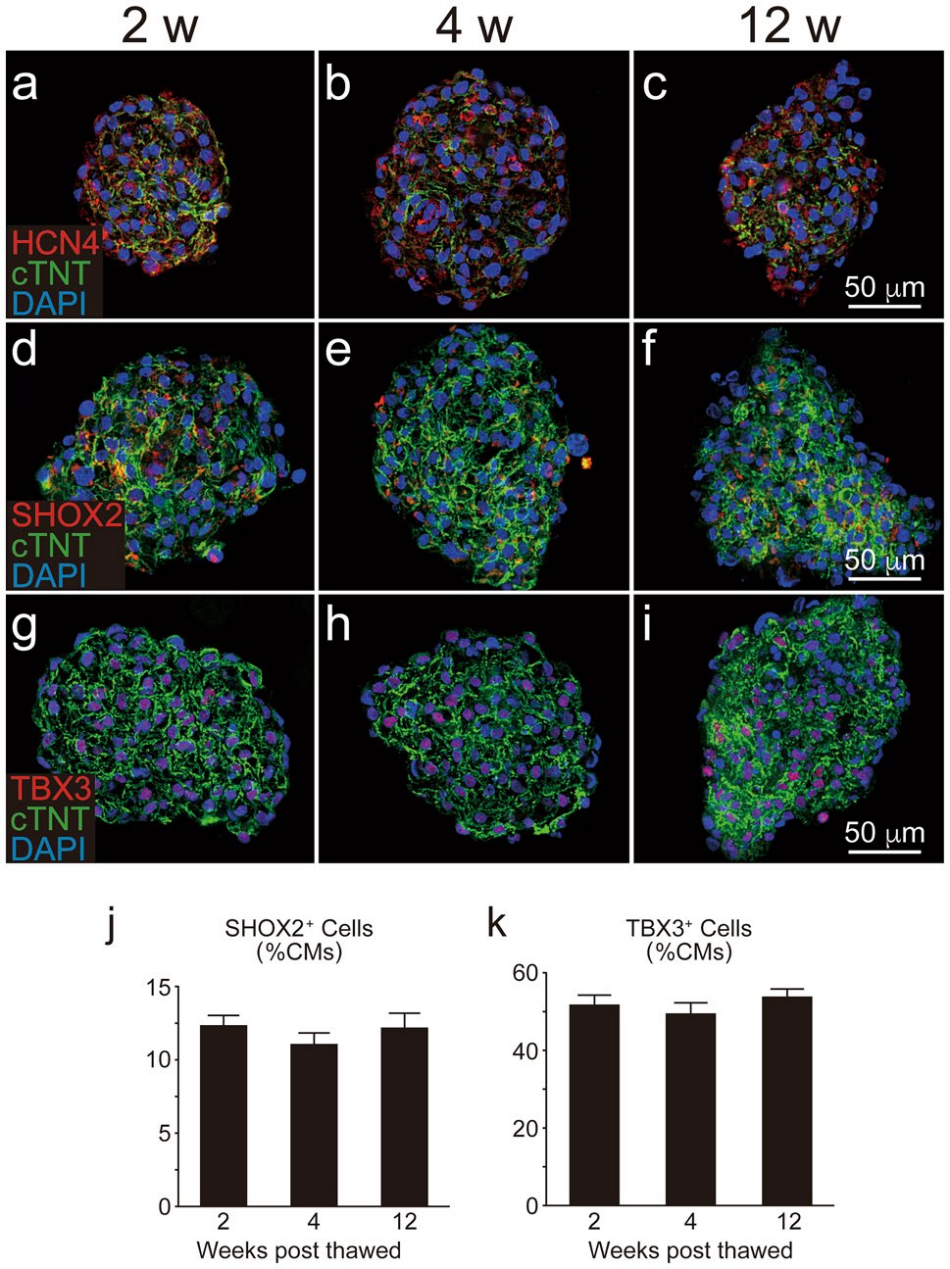
Chronological expression of nodal markers in human ES cell-derived cardiomyocytes (hESC-CMs) on three-dimensional cultures. hESC-CMs were thawed to form cardiac spheroids and cultured. Histological analysis was performed at the same time-points. (**a**–**c**) HCN4 (red) and cTNT (green). (**d**–**f**) SHOX2 (red) and cTNT (green). (**g**–**i**) TBX3 (red) and cTNT (green). (**j**,**k**) Fractions of SHOX2^+^ and TBX3^+^ cardiomyocytes, respectively (n = 3 per group). Data represent mean ± SEM.

To elucidate the chronological alterations of hESC-CMs in vitro, we thawed, re-plated, and cultured the dispersed hESC-CMs for an additional 4–6 days (0 w; n = 3), 2 weeks (2 w; n = 3), 4 weeks (4 w; n = 3), or 12 weeks (12 w; n = 3) (i.e., a total 24–26, 34, 48, or 104 culturing days, SI Fig. S1 online). Consistent with the in vivo outcome, the expression of MLC2A was decreased and that of MLC2V was increased over time (SI Fig. S3a–h online). Unlike the in vivo outcome, however, the fraction of SHOX2 positive cells was not decreased (Fig. 5e–h,m) although the expressions of TBX3 and HCN4 was (Fig. 5a–d,i–l,n). Given that the environment of dispersed cardiomyocytes on two-dimensional culture differs entirely from that of the in vivo heart, we next created and cultured hESC-CM spheroids^15^, in which cardiomyocytes three-dimensionally contact each other. In this three-dimensional culture condition, the expressions of HCN4, SHOX2, and TBX3 were not altered over time (Fig. 6a–k).

### Decreased proliferative capacity of grafted nodal-like cardiomyocytes

**Figure 7:**
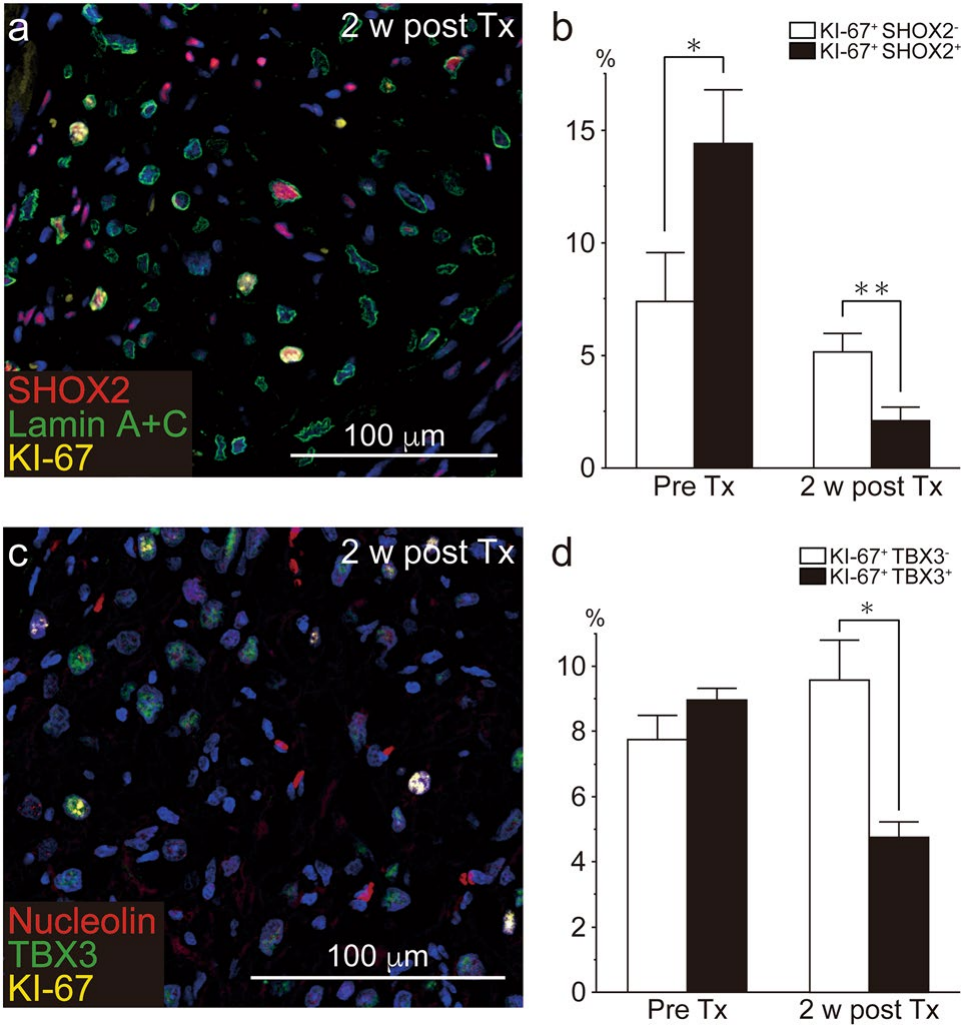
Proliferative capacity of nodal-like cardiomyocytes. Human ES cell-derived cardiomyocytes (hESC-CMs) were stained with the nodal markers SHOX2 or TBX3 and with the proliferative cell marker, KI-67 prior to cell transplantation (Pre-TX; i.e., in vitro) and 2 weeks post transplantation (i.e., in vivo). (**a**) SHOX2 (red), Lamin A+C (green), and KI-67 (yellow) staining in the grafted cardiomyocytes at 2 weeks post transplantation. (**b**) Percentage of KI-67^+^ cells in either SHOX2^−^ (white box) or SHOX2^+^ (black box) cardiomyocytes (Pre-Tx; n = 3, 2 weeks post Tx; n = 5). Data represent mean ± SEM. *P = 0.0479, **P = 0.0472 by Mann–Whitney U test. (**c**) KI-67 (red) and TBX3 (green) staining in the grafted cardiomyocytes at 2 weeks post transplantation. (**d**) Percentage of KI-67^+^ cells in either TBX3^-^ (white box) or TBX3^+^ (black box) cardiomyocytes (Pre-Tx; n = 3, 2 weeks post Tx; n = 5). Data represent mean ± SEM. **P = 0.0090 by Mann–Whitney U test.

We further investigated the mechanisms by which nodal-like graft cardiomyocytes were decreased in vivo. First, we evaluated the effect of apoptosis toward decreasing the fraction of graft nodal-like cells; however, no or few apoptotic TUNNEL-positive cells were observed among either nodal-like or working-type cardiomyocytes (data not shown), indicating that cell apoptosis was extremely unlikely to represent the mechanism by which the ratio of cardiomyocyte fractions changed. Considering that previous studies showed that grafted cardiomyocytes proliferated in vivo^13,14^, we next counted the number of KI-67 positive proliferating graft cells at 2 weeks post transplantation. The KI-67-positive cell fractions in SHOX2-positive graft cells (Fig. 7a), and KI-67-positive cells in TBX3-positive graft cells (Fig. 7c), were significantly lower than that of other KI-67-positive cardiomyocytes (Fig. 7b,d). We further compared the fractions containing the late cytokinesis marker, Anillin-positive cells between nodal-like cells and other cardiomyocytes, however, since Anillin is extremely rarely expressed, we did not observe any statistically meaningful differences (SI Fig. S6 online).

### Gene expression as determined by RNA-sequencing (RNA-Seq) analysis supports the immunochemical outcomes

**Figure 8:**
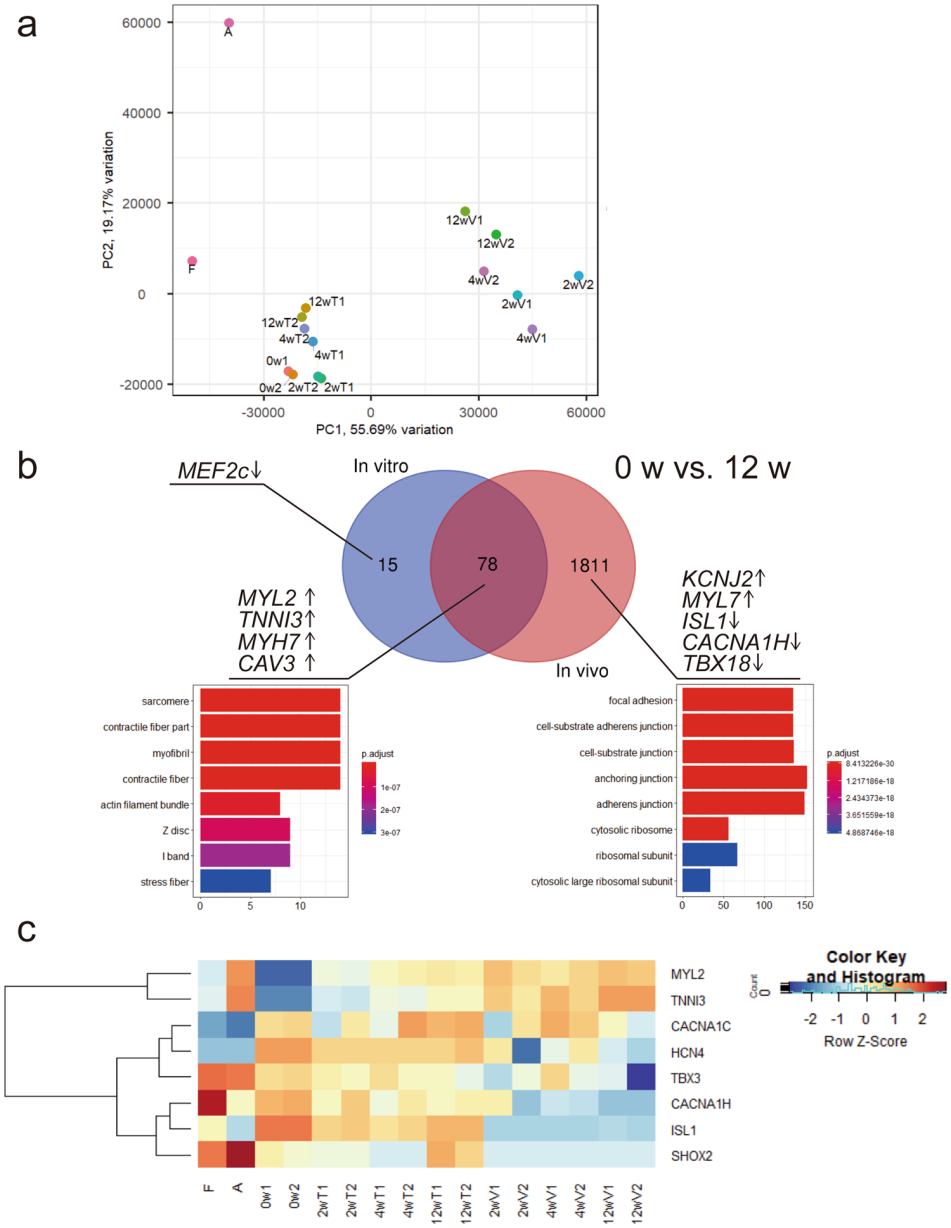
RNA-sequencing analyses of in vitro and in vivo samples. (**a**) Principal-component analysis (PCA) plots with the computation of the closest neighbouring subpopulations. *F* fetal heart, *A* adult heart, *0 w* harvested human ES cell-derived cardiomyocytes (hESC-CMs) at differentiation day 20. *2, 4,* *12 wT* in vitro samples cultured for 2, 4, and 12 weeks, respectively, *2, 4, and 12 wV* in vivo samples of 2, 4, and 12 weeks following transplantation, respectively. (**b**) Comparative expression analysis of in vitro and in vivo enriched genes after 12 weeks over that of pre-treatment (0 week). Venn diagram showing overlap of in vitro and in vivo samples using a false-discovery rate cutoff of 0.01. The top eight enriched gene ontology terms are shown for each group. See also SI Table S2. (**c**) Heatmap showing time course expression of cardiac maturation and pacemaker-related genes. See also SI Figure S7.

We performed RNA-Seq analysis to achieve a detailed understanding of the genetic programmes after transplantation of hESC-CMs. We obtained RNA from both in vitro cell preparations and in vivo engrafted cells in uninjured hearts. The in vivo samples were collected from the graft area using laser microdissection and the reads arising from human cells, which were classified using Xenome software, were analysed^16^. Principal-component analysis (PCA) (Fig. 8a) revealed that substantial gaps existed between the in vitro and in vivo samples. The gene profile of hESC-CMs harvested in vitro at 12 weeks was similar to that of the foetal heart. Hierarchical clustering of in vivo and in vitro samples revealed that biological replicates clustered together, save for in vivo samples at early time-points (SI Fig. S7 online). Differential expression analysis revealed that well-known cardiac maturation markers such as *TNNI3*, *MYL2* (MLC2V), and *MYH7* (β-MHC) were upregulated both in vivo and in vitro at 12 weeks compared to those at 0 week (Fig. 8b, SI Table S2 online). Upregulation of *KCNJ2*, which is primarily responsible for the maintenance of resting membrane potential in ventricular myocytes, as well as downregulation of nodal-cell related genes such as *ISL1*, *CACNA1H,* and *TBX18* were detected only in vivo samples at 12 weeks. Gene Ontology (GO) analysis revealed that in vivo enriched genes were affiliated with GO terms that associated with cell junctions and focal adhesion. The expression of nodal cardiomyocyte-related genes such as *HCN4* and *TBX3* gradually decreased over 12 weeks in vivo but not in vitro (Fig. 8c). Notably, the expression of *SHOX2* and *ISL1*, which encode transcriptional regulators of the pacemaker gene programme^17,18^, were mostly arrested after in vivo transplantation, whereas their expression was retained throughout 12 weeks in vitro culture.

## Discussion

hPSC-CMs are considered to plausibly contribute to the post-transplant VT observed in large animal models through increased automaticity as they exhibit an immature phenotype^19^ with rapid spontaneous beating rate^20,21^ and contain nodal-like cardiomyocytes^22^. Indeed, herein, hESC-derived working-type cardiomyocytes, as well as in vitro nodal-like cardiomyocytes beat at 100 ± 5 and 256 ± 55 beats/min, respectively, which are much faster than the rate of the adult human heart. We also demonstrated that the differential proliferation of these hESC-derived cardiomyocyte fractions also likely led to the transient nature of the post-transplant VT. We note, however, that although several reports have demonstrated that three distinct subtypes of hPSC-CMs exist^23–25^ including nodal-, atrial-, and ventricular-like cardiomyocytes, we were unable to separate atrial-like from ventricular-like cardiomyocytes by their AP patterns. Therefore, in the present study we divided hESC-CMs into two subtypes, nodal-like and working-type cardiomyocytes^26^.

Numerous markers have been reported for the cardiac conduction system (CCS)^27^ including *HCN4*, *SHOX2*, and *TBX3*. Among these, *SHOX2* was shown to be predominant in the sinoatrial node whereas *TBX3* was shown to be expressed both in the sinoatrial and atrioventricular node^27^; however, all of the markers are in non-CCS tissue^28–30^. To practically identify cardiac nodal lineage among hESC-CMs, we utilised these three markers although their labelled fractions or expression levels were not equivalent.

Consistent with previous findings^8,10^, grafted hESC-CMs showed more mature phenotypes over time in vivo. In addition, we found that the fraction of grafted nodal-like cardiomyocytes did not change until 4 weeks after transplantation, at which time post-transplant VT was frequently observed in previous transplantation studies^8–11^, but eventually decreased significantly at 12 weeks after transplantation. Possible mechanisms for the relative decrease in grafted nodal cardiomyocytes include non-apoptotic cell death and transdifferentiation from nodal-like into working-type cardiomyocytes. Although we cannot exclude these mechanisms, in the current study we demonstrated a decreased proliferative capacity of grafted nodal cardiomyocytes. Consistent with previous findings^8,13,14^, we observed that grafted cardiomyocytes proliferated and tended to become larger. Given that the fraction of nodal-like cardiomyocytes and their proliferative capacity were not necessarily decreased in vitro, both in two-dimensional and three-dimensional culture, the in vivo environment wherein the cardiomyocytes are surrounded by ventricular myocytes may inhibit the proliferation of nodal-like cardiomyocytes. Consistent with this concept, RNA-Seq data also revealed the arrest of nodal gene programmes by transcriptional regulators such as *SHOX2* and *ISL1* only in vivo. Nevertheless, additional studies, such as transplanting hPSC-CMs into atrial tissue, will be required to confirm this speculation.

The current study provides novel insights regarding the application of stem cell therapy for cardiac repair; nevertheless, a fundamental question remains unresolved. Although matured ventricular cardiomyocytes become the predominant subtype in the rat heart among the grafted cardiomyocytes over time, it remains to be determined whether cardiomyocyte immaturity or the existence of nodal-like cardiomyocytes directly leads to post-transplant VT. Transplantation of matured ventricular cardiomyocytes into large animal models, such as pigs or monkeys, in which post-transplant VT can be detected would therefore be worthwhile.

In conclusion, we found that hESC-CMs consisted of nodal-like and working-type cardiomyocytes. Grafted nodal-like cardiomyocytes transiently engrafted in the rat heart but did not survive over the long-term, which may explain the occurrence of transient post-transplant VT; however, further study will be required to confirm this mechanism.

## Methods

### Cell preparation

An undifferentiated embryonic stem cell line, H9, was cultured using Essential 8 (E8) medium (Thermo Fisher) with feeder SNL cells. hESC-CMs were differentiated in accordance with our previously reported protocol^31^. Briefly, cultured undifferentiated ES cells on SNL feeder cells were passaged and re-plated on Matrigel (Corning)-coated culture dishes and cultured in E8 medium for another few days. When the cells reached 90% confluency, E8 medium was supplied with 1 μM of the Wnt activator CHIR99201 (Sigma-Aldrich). The next day (day 0), E8 medium was changed to cardiac differentiation medium (RPMI 1640 plus B27 supplement minus insulin (Gibco) plus l-glutamine with added activin A (100 ng/mL, R&D) and Matrigel. On day 1, bone morphologic protein 4 (BMP4; 10 ng/mL, R&D) and CHIR99201 were added, followed by addition of the Wnt inhibitor XAV939 (1 μmol/L, Sigma-Aldrich) on day 3–4. After day 7, the medium was changed to RPMI 1,640 with B27 supplement (Gibco) and replaced every other day. The cells were heat-shocked at 43 °C for 30 min and cryopreserved on day 20. Cardiac purity was determined by flow cytometry (BD Biosciences) by staining against cTNT (Thermofisher, clone 13-11) or a mouse immunoglobulin G1 (IgG1) κ isotype control (BioLegend, clone MG1–45), followed by anti-mouse IgG1 conjugated with phycoerythrin. Prior to cell transplantation, 2 × 10^7^ cells were thawed and diluted with 70 μL of pro-survival cocktail as previously reported^3,32^.

### Spheroid formation of hESC-CMs

Cryopreserved hESC-CMs were thawed and re-plated in 24-well plates (Elplasia RB 500 400 NA 24; Kuraray). Spheroids were spontaneously formed and cultured until their specific endpoints (i.e. 2, 4, or 12 weeks). Half of the medium was changed every 3 days for 2, 4 or 12 weeks.

### Electrophysiological analysis of hESC-CMs by patch-clamp

Cryopreserved hESC-CMs were thawed, re-plated, and cultured for an additional 4 days for in vitro electrophysiological analysis. To examine the autonomic beating rate, the maximum dV/dt of depolarisation (dV/dt_max_), and action potential duration at 50% and 90% repolarisation (APD_50_, and APD_90_, respectively), of spontaneous action potentials were recorded using a ruptured whole-cell patch-clamp technique in the current-clamp mode at 35–36 °C using a patch-clamp amplifier (Axopatch 200B, Molecular Devices) and sampled at 5 kHz after being low-pass-filtered at 2 kHz^33^. Patch pipettes (7–8 MΩ) were fabricated from borosilicate glass capillaries (Kimax-51, Kimble Glass) and coated with Sylgard 184 (Dow Corning Toray Co.). Series resistance was always kept below 20 MΩ. Action potentials were measured using an intracellular solution containing (mmol/L): 130 potassium gluconate (Wako), 10 KCl (Wako), 5 NaCl (Wako), 1 MgCl_2_ (Wako), 0.1 EGTA (Dojindo), 0.1 Mg ATP, and 10 HEPES (Dojindo) [pH 7.2 with KOH (Wako)]. The extracellular bath solution contained (mmol/L): 136.5 NaCl, 5.4 KCl, 1.8 CaCl_2_ (Wako), 0.53 MgCl_2_, 5.5 HEPES, and 5.5 glucose (Wako) (pH 7.4 with NaOH).

### Animal surgeries

Based on the national regulations and guidelines, all experimental procedures were reviewed by the Committee for Animal Experiments and finally approved by the president of Shinshu University. Ten- to twelve-week-old male athymic rats (F344/NJcl-rnu/rnu, CLEA Japan) were anaesthetised via an intraperitoneal injection of 0.15 mg/kg medetomidine, 2 mg/kg midazolam, and 2.5 mg/kg butorphanol. The animals were intubated and mechanically ventilated with 2.5% sevoflurane. Following left intercostal thoracotomy, the heart was exposed. A total of 2 × 10^7^ hESC-CMs diluted with pro-survival cocktail were directly injected at two sites of the anterior wall of the rat hearts using a 29 gauge injection needle. Subcutaneous meloxicam was routinely administered to provide postoperative pain relief.

The rat myocardial infarction model was produced via ligation of the left anterior descending artery with a 6-0 braided silk (Natsume Seisakusho) below the left atrial appendage level. On day 7 after induction of myocardial infarction, hESC-CMs were injected in the same way as described above.

### Echocardiography

Echocardiography was performed 1 week after myocardial infarction (pre-Tx), as well as 4, 8, and 12 weeks after cell transplantation using transthoracic echocardiography (Vevo2100; Primetech) with a 30-MHz transducer (MX400). At each time point, the animals were anaesthetised with 3% isoflurane, and the left-ventricular end-diastolic dimension (LVEDD), left-ventricular end-systolic dimension (LVESD) and heart rate were measured. Fractional shortening (FS) was calculated according to Eq. (1):

1 $$FS=100 \times \left(\frac{LVEDD-LVESD}{LVEDD}\right)$$

All measurements were taken over three cardiac cycles, which were then averaged. An operator blinded to the study groups performed all measurements.

### Histology and immunocytochemistry

Cultured hESC-CMs were fixed with 2% paraformaldehyde for 10 min. After permeabilisation and blocking of nonspecific binding by 1.5% goat serum with 0.1% Triton-X 100 (MP Biomedicals) in phosphate buffered saline, cells were stained with primary antibodies followed by species corresponding secondary antibodies.

Cultured hESC-CM spheroids were fixed with 4% paraformaldehyde for 24 h. After sucrose replacement, spheroids were embedded in OCT-embedded compound (Sakura Finetek Japan) and stored at − 80 °C. Tissues were sectioned at a thickness of 10 µm using a Cryostat (Leica). After permeabilisation and blocking of nonspecific binding, serial sections were stained with primary antibodies followed by appropriate secondary antibodies.

For in vivo histological analysis, the rats were sacrificed at 2, 4, or 12 weeks post cell transplantation. The rat hearts were collected, sliced at 2 mm thickness, fixed with 4% paraformaldehyde for 24 h, embedded with paraffin, and eventually sectioned at 4 μm thickness using a sliding microtome. After quenching of endogenous peroxidase, antigen retrieval with pH 6.0 citrate buffer, and blocking of nonspecific binding by 1.5% goat or donkey serum in phosphate buffered saline, sections were incubated with primary antibodies followed by species corresponding secondary antibodies.

Antibodies utilised in this study are listed in SI Table S1 online.

### Quantification of stained sections

The number of cells in the graft area was quantified by counting the number of stained nuclei (SHOX2, TBX3, KI-67, Anillin, Nucleolin and Lamin A+C) or cytoplasm (MLC2A and MLC2V) using ImageJ (NIH) software. Graft areas were measured using NDP.view2 (Hamamatsu).

### RNA-Seq

For collection of in vitro samples, cells were detached and total RNA was extracted using ISOGEN (Nippon Gene), phenol, and chloroform. Human fetal heart RNA (Clontech 636583) and human adult heart RNA (Clontech 636532) were purchased from TaKaRa Bio. For in vivo samples, rat hearts of each time point were collected, sliced as described above, and immediately embedded in an OCT-embedding compound and stored at − 80 °C. Tissues were sectioned at a thickness of 10 µm using a Cryostat (Leica) and serial sections were stained with hematoxylin and eosin to detect the graft area. The graft areas were captured using a laser microdissection system (Leica) from unstained unfixed specimens attached on membrane-coated slides (Leica 11600289). Total RNA was extracted using the Arcturus PicoPure RNA Isolation Kit (Thermo). cDNA was synthesised using the SMART-Seq v4 Ultra Low Input RNA Kit for Sequencing (TaKaRa Bio). Library preparations were conducted using the Nextera DNA Library Prep Kit and subjected to sequencing on a NovaSeq 6000 platform (Illumina).

RNA-Seq reads were trimmed using Trimmomatic (v0.39) with parameters of SLIDINGWINDOW:10:30^34^. All the samples were separated into human and rat reads using Xenome (v1.0.0)^15^. Reads classified as human were mapped to the hg38 reference using STAR (v2.7.2a)^35^ and a gene count matrix was generated using featureCounts (v1.6.4)^36^. Differential expression analysis was performed using the edgeR package, with trimmed mean of M values (TMM) normalisation^37^. PCA was performed and a plot generated using PCAtools package (https://github.com/kevinblighe/PCAtools), with transcripts per million (TPM) normalisation. GO enrichment was performed using the clusterProfiler package^38^.

### Statistical analysis

Statistical significance (P < 0.05) was calculated using a two-sided Student’s t test or Mann–Whitney U test to compare two groups. To compare more than two groups, analysis of variance (ANOVA) followed by Tukey’s post hoc test was performed. All values are described as means, and error bars in the figures represent standard error of the mean. All statistical analysis was performed using JMP software.

## Supplementary information

Supplementary information

## Data Availability

RNA-Seq data were deposited in the NCBI’s Gene Expression Omnibus (GEO series accession number GSE137255).
