# Supplementary material for: Increased predominance of the matured ventricular subtype in embryonic stem cell-derived cardiomyocytes in vivo
Source: Sci Rep. 2020 Jul 17;10:11883. doi: 10.1038/s41598-020-68373-9 (PMC7368005; doi:10.1038/s41598-020-68373-9)
Supplement: Supplementary file 1 — Supplementary information [file 41598_2020_68373_MOESM1_ESM.docx]

**Increased Predominance of the Matured Ventricular Subtype**

**in Embryonic Stem Cell-Derived Cardiomyocytes *In Vivo***

**Hajime Ichimura,^1,2,3^ Shin Kadota,^1,2,^* Toshihide Kashihara,^4,5^ Mitsuhiko Yamada,^5^ Kuniaki Ito,^1^ Hideki Kobayashi,^1,2,6^ Yuki Tanaka,^1,2^ Naoko Shiba,^1,2,7^ Shinichiro Chuma,^8^ Shugo Tohyama,^9^ Tatsuichiro Seto,^3^ Kenji Okada,^10^ Koichiro Kuwahara,^2,6^ and Yuji Shiba^1,2,^***

^1^Department of Regenerative Science and Medicine, Shinshu University School of Medicine

^2^Institute for Biomedical Sciences, Shinshu University

^3^Division of Cardiovascular Surgery, Department of Surgery, Shinshu University School of Medicine

^4^Department of Cell Biology and Molecular Medicine, Cardiovascular Research Institute, Rutgers New Jersey Medical School

^5^Department of Molecular Pharmacology, Shinshu University School of Medicine

^6^Department of Cardiovascular Medicine, Shinshu University School of Medicine

^7^Department of Pediatrics, Shinshu University School of Medicine

^8^Department of Development and Differentiation, Institute for Frontier Life and Medical Sciences, Kyoto University

^9^Department of Cardiology, Keio University

^10^Department of Surgery, Division of Cardiovascular Surgery, Kobe University Graduate School of Medicine

*Corresponding: shinkadota@shinshu-u.ac.jp (S.K.) or yshiba@shinshu-u.ac.jp (Y.S.)

**
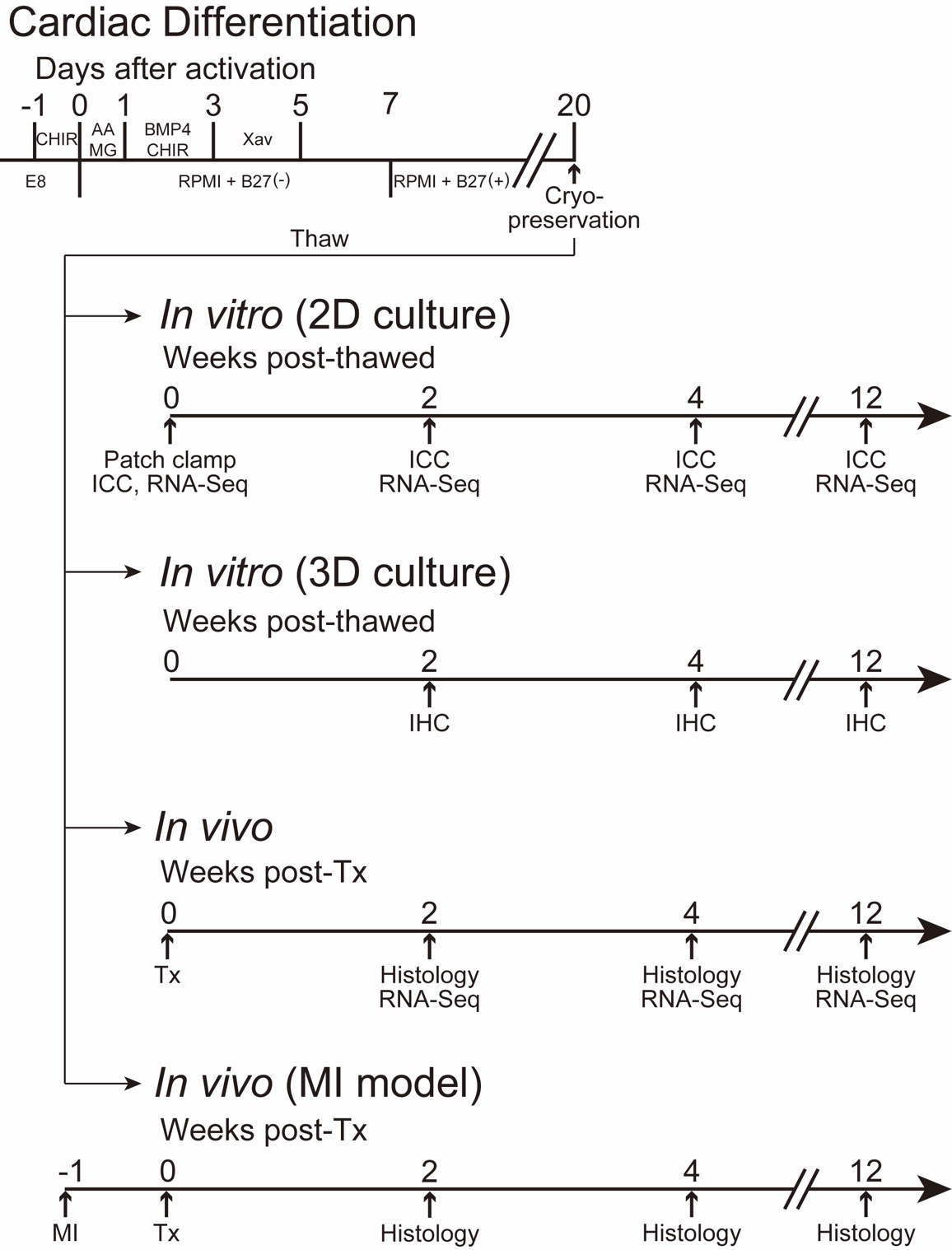
**

**Supplementary Figure S1. Schematic View of the *In Vitro* and *In Vivo* Study Design.**

Undifferentiated human embryonic stem cells (hESCs) (day 0) were transdifferentiated into cardiomyocytes (CMs) based on a previously reported monolayer-based direct differentiation protocol until day 20. Then, the hESC-CMs were cryopreserved and thawed for *in vitro* and *in vivo* experiments using the same batch of hESC-CMs. Note that time scales in weeks for *in vitro* and *in vivo* experiments correspond to each other. E8, Essential 8 medium; CHIR, CHIR99201; AA, activin A; MG, Matrigel; Xav, Xav939; ICC, immunocytochemistry; RNA-Seq, RNA sequencing; Tx, transplantation; MI, myocardial infarction.

**
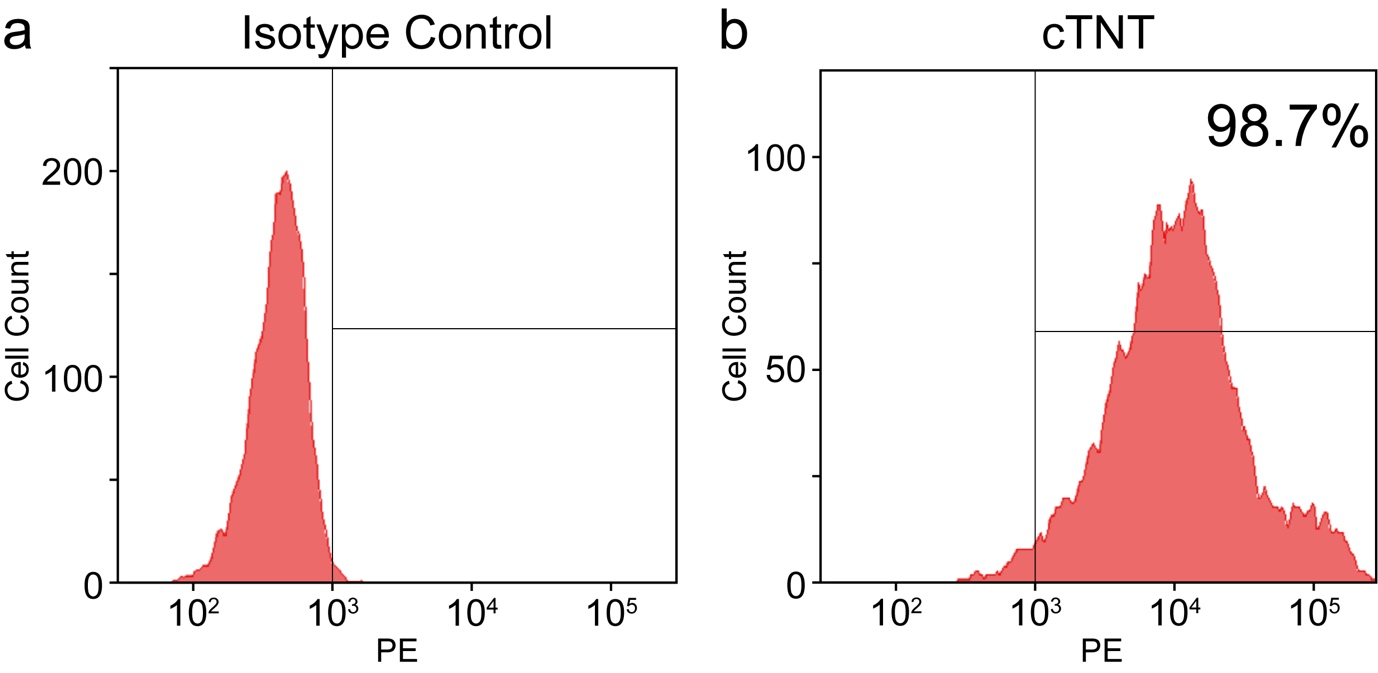
**

**Supplementary Figure S2. Flow Cytometric Analysis for Cardiac Purity of Human Embryonic Stem Cell-derived Cardiomyocytes (hESC-CMs) as Determined by Cardiac Troponin T (cTNT) Staining.**

On day 20 prior to cryopreservation, a subset of hESC-CMs was fixed and stained with cTNT to determine cardiac purity. (**a**) Isotype control. (**b**) Cardiac purity of hESC-CMs used in this study was 98.7%. PE, phycoerythrin.

**
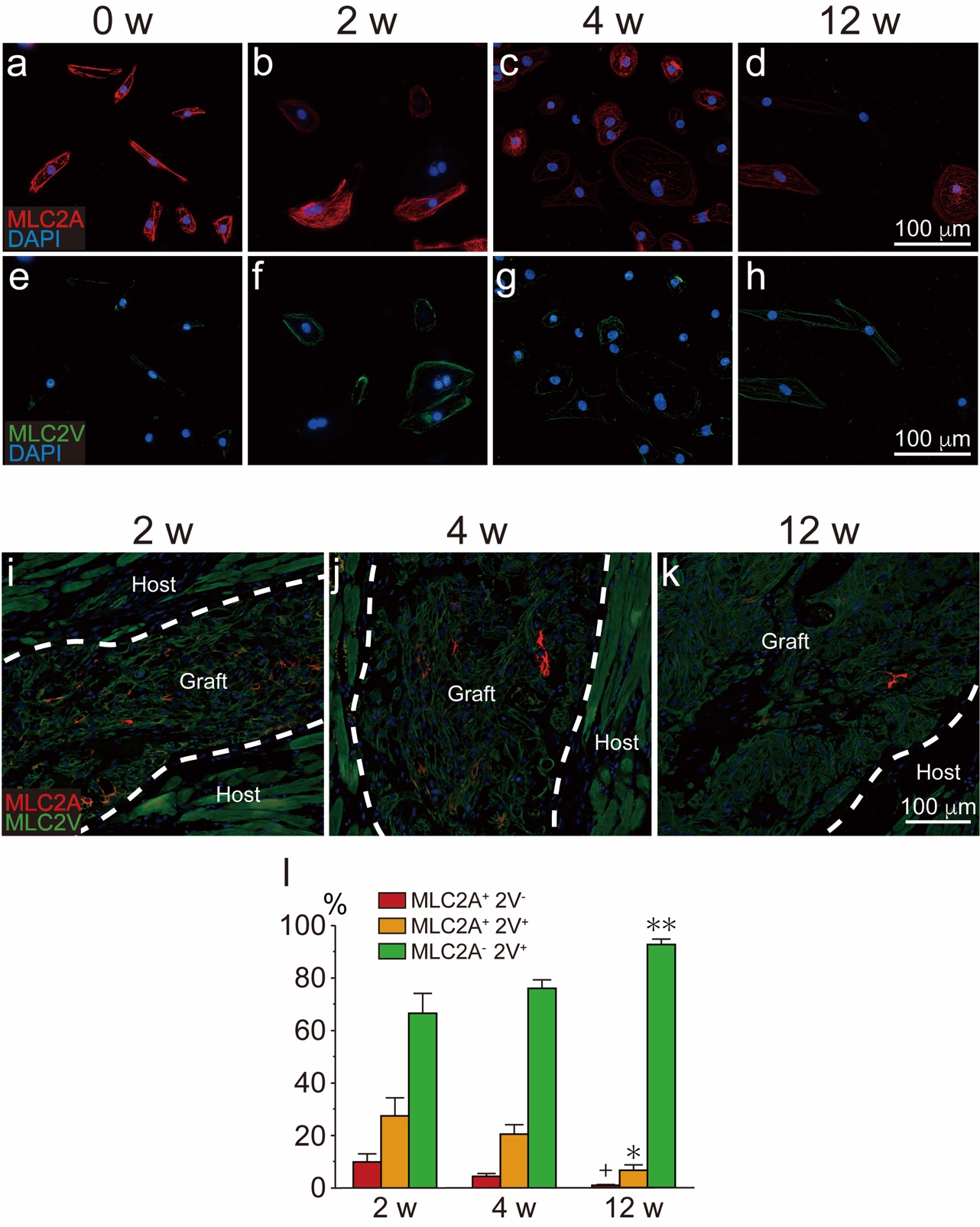
**

**Supplementary Figure S3. Chronological Expression of MLC2a and MLC2b in Human Embryonic Stem Cell-derived Cardiomyocytes (hESC-CMs) *In Vitro* and *In Vivo*.**

Expression of MLC2A (**a-d**) and MLC2V (**e-h**) *in vitro*. (**i-k**) Expression of MLC2A (red) and MLC2V (green) *in vivo*. Colocalization of the two antigens is expressed in orange. (**l**) Quantitative measurement of the area expressed in either MLC2A^+^, MLC2A^+^/MLC2V^+^, or MLC2V^+^ (n = 5 per group). Data represent mean ± SEM. +P = 0.0058 vs. 2 w, *P = 0.0134 vs. 2 w, **P = 0.0048 vs. 2 w by ANOVA with Tukey’s post hoc test.

**
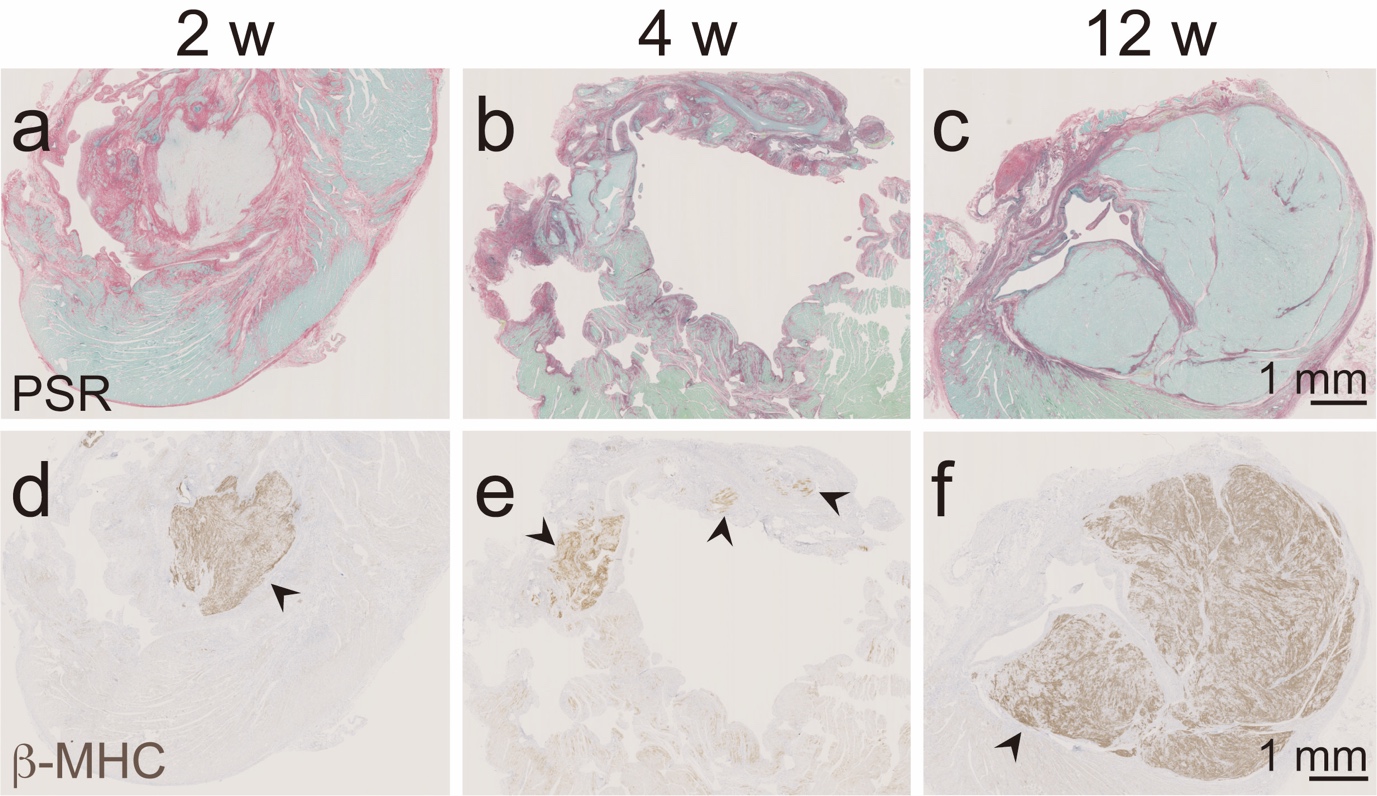
**

**Supplementary Figure S4. Engraftment of Human ES Cell-derived Cardiomyocytes (hESC-CMs) in Injured Athymic Rat Hearts.**

One week after myocardial infarction, hESC-CMs were injected directly into athymic rat hearts and histological analysis was performed at 2, 4, and 12 weeks post-transplantation. (**a-c**) Picro-sirius red (PSR) staining of grafted hESC-CMs in the host hearts. (**d-f**) Human grafted cardiomyocytes identified by the cardiac marker, β-myosin heavy chain (β-MHC, arrowheads). Note that β-MHC was exclusively expressed in grafted human cardiomyocytes and not in host rat cardiomyocytes.

**
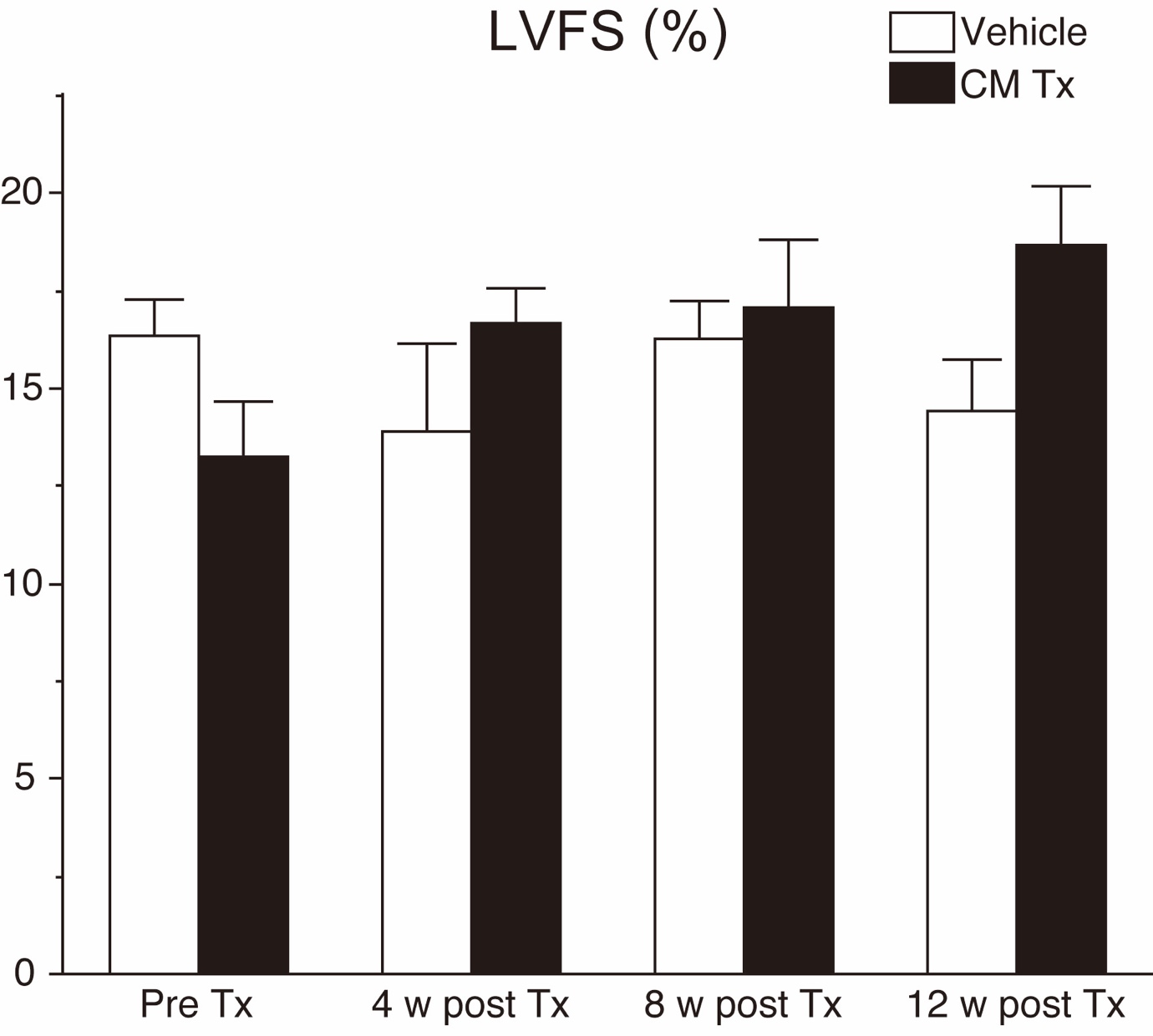
**

**Supplementary Figure S5. Fractional Shortening of Left Ventricle by Echocardiography.**

Echocardiography was performed and fractional shortening of left ventricle (LVFS) was calculated before cell transplantation (Pre-Tx), as well as 4 weeks (4 w post-Tx), 8 weeks (8 w post-Tx), and 12 weeks after cell transplantation (12 w post-Tx). Vehicle indicates recipient group that underwent myocardial infarction treated with cell-free vehicle injection. There were no significant differences observed between experimental groups and time points. Data represent mean ± SEM. CM, cardiomyocyte; Tx, transplantation.

**
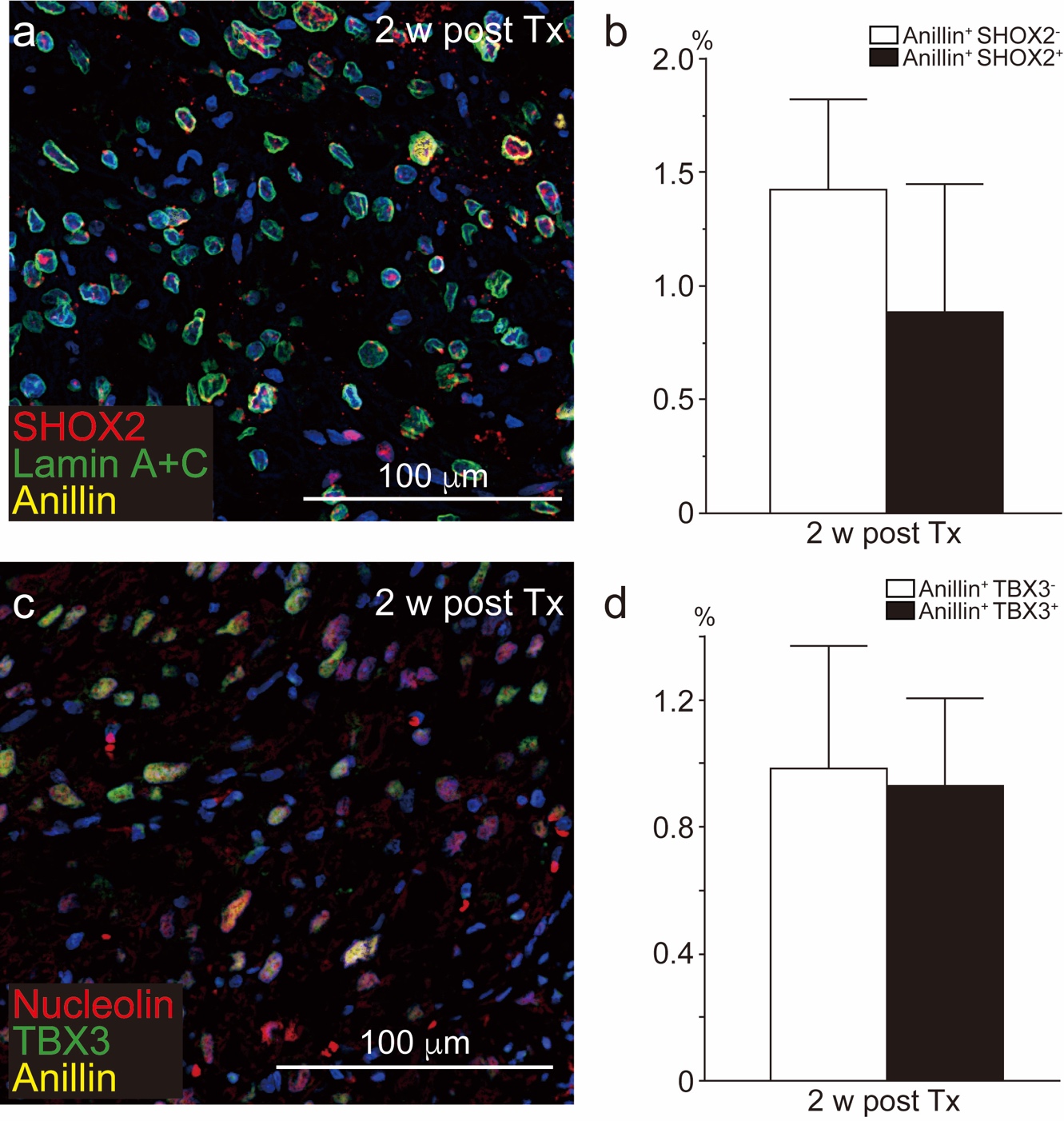
**

**Supplementary Figure S6. Proliferative Capacity of Nodal-like Cardiomyocytes.**

Grafted cardiomyocytes were stained with the nodal markers SHOX2 or TBX3, and with the cytokinesis marker, Anillin at 2 weeks post transplantation (2 w post Tx). (**a**) SHOX2 (red), Lamin A+C (green), and Anillin (yellow) staining in the grafted cardiomyocytes. (**b**) Percentage of Anillin^+^ cells in either SHOX2^-^ (white box) or SHOX2^+^ (black box) cardiomyocytes (n = 4). Data represent mean ± SEM. (**c**) Nucleolin (red), TBX3 (green), and Anillin (yellow) staining in the grafted cardiomyocytes. (**d**) Percentage of Anillin^+^ cells in either TBX3^-^ (white box) or TBX3^+^ (black box) cardiomyocytes (n = 4). Data represent mean ± SEM.

**
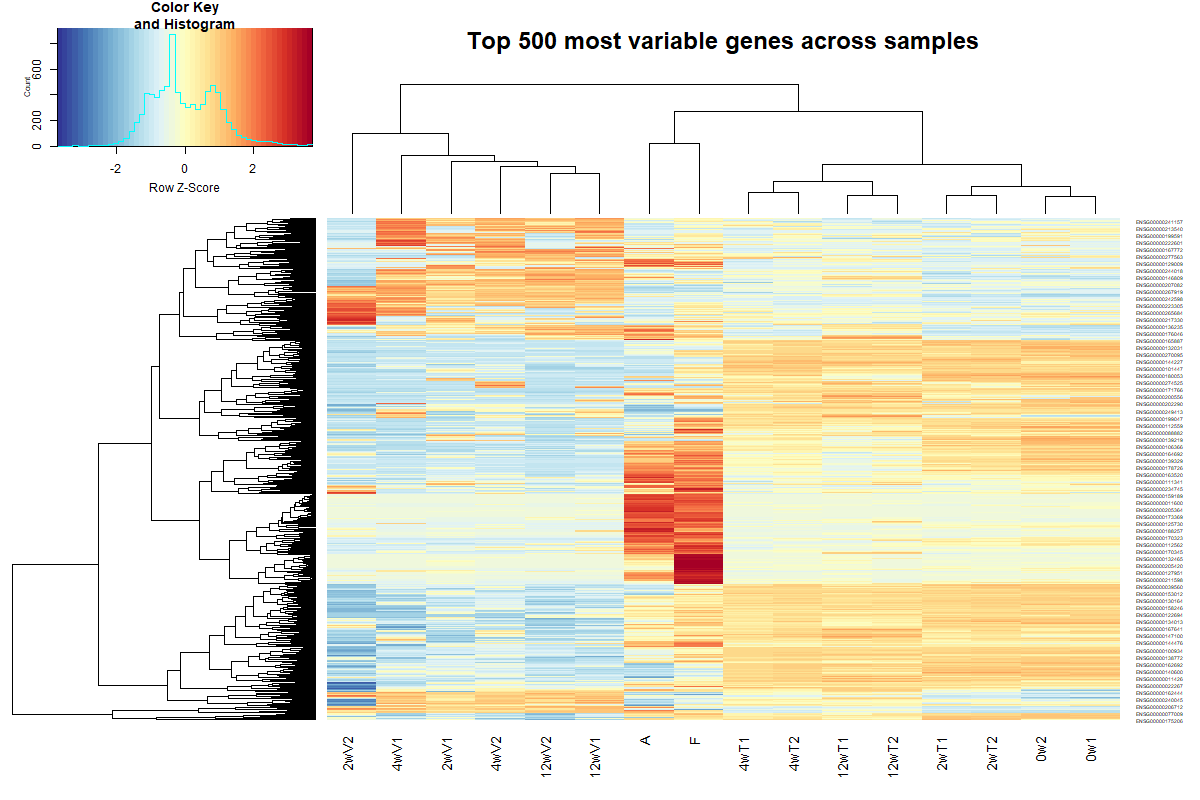
**

**Supplementary Figure S7. Hierarchical Bi-clustering of *In Vivo* and *In Vitro* Samples.**

The top 500 most variable genes across all samples were used for clustering.

**Supplementary Table S1. Antibodies Used for Immunostaining.**

**
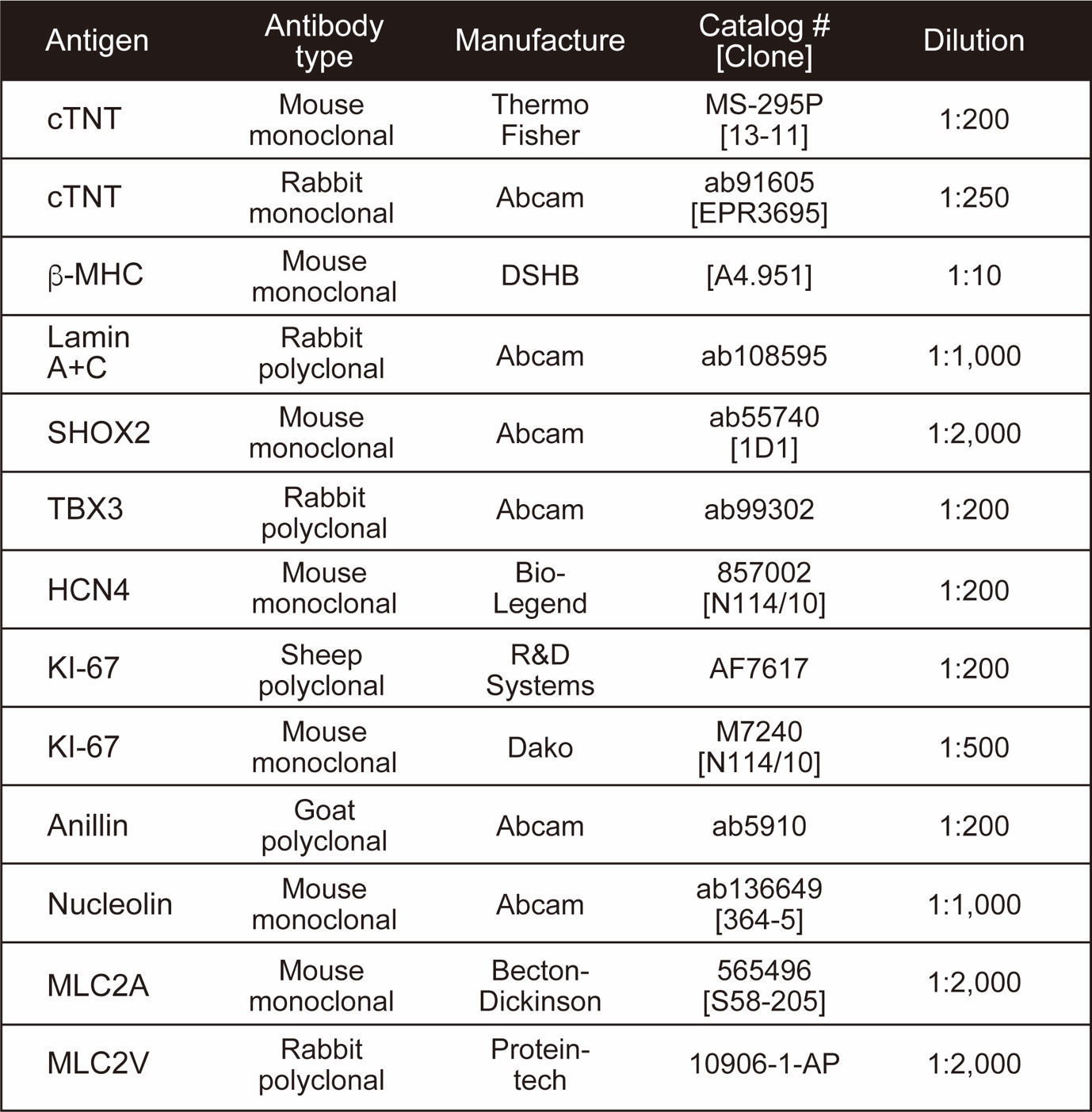
**

**Supplementary Table S2. Fold Change (FC), P-value and False Discovery Rate (FDR) of Critical Genes.**

|  | In vitro |  |  | In vivo |  |  |
| --- | --- | --- | --- | --- | --- | --- |
|  | logFC | P-Value | FDR | logFC | P-Value | FDR |
| MYL2 | 4.150928 | 1.66E-10 | 3.16E-06 | 5.478267 | 1.10E-11 | 7.39E-08 |
| TNNI3 | 3.442319 | 1.23E-08 | 3.21E-05 | 6.086173 | 4.82E-11 | 2.27E-07 |
| MYH6 | -3.00288 | 2.11E-09 | 1.02E-05 | 1.226428 | 2.86E-05 | 0.001082 |
| MYH7 | 2.434334 | 2.29E-07 | 0.000283 | 1.439673 | 4.64E-05 | 0.001497 |
| CAV3 | 5.124208 | 3.48E-05 | 0.008811 | 4.462448 | 0.000104 | 0.002443 |
| MEF2C | -2.41427 | 7.29E-06 | 0.003069 |  |  |  |
| KCNJ2 |  |  |  | 8.296593 | 2.67E-07 | 5.49E-05 |
| MYL7 |  |  |  | 1.154484 | 4.63E-07 | 7.57E-05 |
| ISL1 |  |  |  | -7.00055 | 0.000172 | 0.003462 |
| CACNA1H |  |  |  | -11.7782 | 4.15E-05 | 0.001386 |
| TBX18 |  |  |  | -9.5156 | 0.000299 | 0.004984 |
